# MS can be considered a primary progressive disease in all cases, but some patients have superimposed relapses – Commentary

**DOI:** 10.1177/13524585211010070

**Published:** 2021-04-20

**Authors:** K Schmierer, G Giovannoni

**Affiliations:** Centre for Neuroscience, Surgery and Trauma, Barts and the London School of Medicine and Dentistry, Blizard Institute, Queen Mary University of London, London, UK/Clinical Board Medicine (Neuroscience), The Royal London Hospital, Barts Health NHS Trust, London, UK; Centre for Neuroscience, Surgery and Trauma, Barts and the London School of Medicine and Dentistry, Blizard Institute, Queen Mary University of London, London, UK/Clinical Board Medicine (Neuroscience), The Royal London Hospital, Barts Health NHS Trust, London, UK/Preventive Neurology Unit, Wolfson Institute of Preventive Medicine, Barts and the London School of Medicine and Dentistry, Queen Mary University of London, London, UK

The phrase *all cases* in the title of this debate appears to have prompted two quite different perspectives on the subject. Antonio Scalfari emphasises similarities between progressive multiple sclerosis (PMS) and relapsing multiple sclerosis (RMS) largely based on epidemiology and pathology data and the observation that even in people with RMS the lion’s share of disease progression appears to occur independent of relapses (PIRA).^1^ Anne Cross and Robert Naismith, on the other hand, challenge the notion of MS as an invariably progressive disease altogether, arguing that rather than PMS or RMS a significant minority of people with MS experiences benign multiple sclerosis (BMS). Although both exciting in their own right, these are essentially two different debates.

Scalfari rightly points at a lack of universally accepted phenotype boundaries or surrogate marker(s) separating RMS and PMS. We hesitate to sign up to the claim of *objectivity* of a new definition recently proposed for secondary PMS.^2^ Even a definition based on analysis of a large sample of prior definitions, put forward by an equally large number of investigators, is going to be time-limited and within the reference frame of the current scientific paradigm.^3^ This does not diminish the value of an evidence-based consensus about how to define secondary PMS for use in contemporary clinical trials.^2^ Scalfari underpins his case further by pointing out that MS lesion pathology differs in quantity much rather than quality across phenotypes.^4^ While such single time point *post-mortem* observations could be challenged on account of their cumulative nature with pathology accrued over decades, the recent discovery that even in RMS, and arguably even in radiologically isolated syndrome,^5^ worsening of disability appears largely independent of relapses further supports the hypothesis of MS as a progressive disease from onset.^1^ More research is evidently needed to explore the mechanisms underlying PIRA. Based on pathology^6^ and genetic data,^7^ and the response to highly effective disease-modifying treatment (DMT)^8^ it appears unlikely that PIRA is a substrate of MS being a primary neurodegenerative disease, with inflammatory demyelination being a secondary, perhaps not even relevant phenomenon. Processes downstream the inflammatory demyelinating injury, however, evidently play an important role for the neuro-axonal damage and loss, and resulting disability accrual.^9^

Where does this leave the discussion about disease progression affecting *all people* with MS? Although definitions vary, we agree with Cross and Naismith that BMS does exist.^10^ Thus, we could simply state: they won the debate! However, the key issue with BMS is recognising it at the point where it truly matters, that is, when decisions about management, including DMT and wider concerns such as family and career planning are first being addressed. The odds of 1/5 (at best!) having benign disease at diagnosis will reassure neither the patient nor their care team. While on a group level, clinical and magnetic resonance imaging (MRI) indices may make BMS more or less likely, these indices are of little help in clinical decision-making. One could also argue that treating MS with DMT increases your odds of having BMS. Further research is required to enable accurate individual risk profiling allowing, for example, a watch-and-wait approach regarding DMT.
